# LLMs are ideological chameleons: personalized echo chambers in the Brazilian political context

**DOI:** 10.1038/s41598-026-52105-6

**Published:** 2026-05-21

**Authors:** Anderson Luis Bento Soares, Bruno Antonio Veiga de Almeida, Leonardo Nascimento Ferreira, Anderson Rocha, Ruben Interian, Zanoni Dias

**Affiliations:** Institute of Computing, University of Campinas (Unicamp), Campinas, SP 13083-852 Brazil

**Keywords:** Large language models, Political bias, Polarization, Sycophancy, Ideological adaptation, Politics and international relations, Science, technology and society

## Abstract

Users are increasingly relying on Large Language Models (LLMs) for political questions, seeking advice and interpreting public debates. This raises a key concern: do LLMs behave as neutral tools, or do they adapt to users’ ideology and reinforce existing polarization? We address this question within the Brazilian context using a new benchmark that probes ideological chameleon behavior in 21 LLMs. We construct opposing pairs of political statements on salient topics (e.g., welfare, security, democratic institutions, environment, and others) and elicit model judgments under three user-position conditions: no context, left-aligned, and right-aligned. To avoid imposing our own ideological taxonomy, we apply a cross-model voting step using independent-judge models and retain only those pairs that are unanimously classified as left versus right. In total, we analyze 47,376 Likert-style responses across models, topics, and hyperparameters (e.g., temperature). We focus on the relative ideological shift induced by user framing. We find that all 21 evaluated models exhibit ideological chameleon behavior to varying degrees. When conditioned by users’ declared leanings, they systematically adjust their stance to align with those views, indicating widespread sycophantic tendencies. These results suggest that many LLMs may function as personalized echo chambers under user framing, with implications for political microtargeting, persuasion, and the governance of AI systems in polarized societies.

## Introduction

Political polarization has intensified in many countries over the past decade, shaping how citizens interpret events, trust institutions, and engage with public debate^1^. Disputes over social welfare, public security, environmental policy, and the role of democratic institutions are increasingly mediated by digital platforms, where ideological validation often takes precedence over scrutiny of evidence^2^. In parallel, large language models (LLMs) have emerged as de facto default options in search engines^3^ and chat-based assistants, offering on-demand summaries of news, explanations of political concepts, and conversational guidance on contentious issues. As these systems move from experimental tools to everyday proxies of information for many users, they are no longer merely generators of text, but potential drivers for how individuals understand and navigate political conflict^4^. In such a context, understanding how LLMs behave when confronted with politically sensitive content goes beyond technical model alignment. It raises broader concerns about their role in already polarized information ecosystems.

Recent work shows that LLMs can actively shape political beliefs. Evidence suggests that messages generated by LLMs can measurably shift attitudes on a range of policy and political issues, including controversial ones, with persuasive effects comparable to human-written arguments^5^. Another work extends this evidence to actual electoral settings, showing that human–AI dialogues advocating for specific candidates in elections in the United States, Canada, and Poland can change vote intentions more than traditional campaign advertisements^4^. Importantly, they also report that, in some conditions, models defending certain candidates produce more factually inaccurate statements while remaining persuasive, indicating that argument quality in practice can trade off against accuracy^4^. In contrast, experimental work in political psychology suggests that when citizens are exposed to large, balanced sets of verifiable facts on both sides of a controversial issue, increased factual knowledge can lead to more moderate, less extreme policy attitudes rather than further polarization^6^. Together, these findings suggest that LLMs can serve as high-capacity engines of political persuasion and that their conversational strategies need not always prioritize factual accuracy when influencing users’ views, even though access to credible, balanced information can reduce extremity.

By applying a new behavioral benchmark to a non-English context, we aim to explore an underrepresented area rather than merely pointing out limitations of existing Anglo-centric benchmarks. For example, when ChatGPT is used to simulate public opinion across countries, its outputs align much more closely with human survey responses in Western, English-speaking, economically developed contexts, especially the United States, than in countries such as Brazil, Japan, and South Africa^7^. These results indicate that current models have limited algorithmic fidelity outside the environments that dominate their training data and highlight the need for culturally and linguistically grounded assessments of LLM behavior. This gap is especially salient for non-English-speaking countries, such as Brazil, where core controversies involve local institutions, cultural references, and historical context that are only weakly represented in global English-language corpora^8^.

In parallel to these cross-country fidelity gaps, another line of work examines the political values embedded in the models themselves. Political Compass–style tests have been adapted to locate successive versions of ChatGPT in an ideological space, finding that they start in the libertarian-left quadrant and gradually move toward a more centrist configuration over time^9^. Complementary analyses based on fine-grained political questionnaires and voting advice tools show that many open-source models tend to lean liberal or progressive on multiple topics and align more closely with left-leaning than right-leaning positions, with bias patterns that depend on geography and language^10,11^. Industry work by OpenAI and Anthropic mirrors these concerns by proposing multi-axis frameworks for political bias and even-handedness in realistic conversations, and by explicitly aiming to steer models toward a notion of neutrality in which objectivity is supposed to remain invariant to the user’s ideological framing^12,13^. Taken together with evidence of country-level disparities^7^, this literature suggests that political behavior in LLMs is both structured and steerable, but unevenly aligned across contexts.

Nevertheless, focusing primarily on aggregate left–right bias and pulling models toward a nominal ideological center does not fully address how LLMs behave in politically charged interactions. In real usage, neutrality involves more than a point on a spectrum. It depends on how models respond when users arrive with strong prior commitments, often in environments saturated with misinformation and competing narratives. Experimental evidence already hints at this tension: in some conditions, models remain persuasive while relying on arguments that can be factually inaccurate^4^. In such cases, a model that appears “less polarized” on average may still contribute to epistemic harm if it selectively omits uncomfortable facts or frames issues in ways that flatter the user’s prior beliefs. Rather than asking only whether models can be steered toward an abstract neutral midpoint, we argue that a crucial dimension of safety lies in their willingness to resist ideological flattery. In politically sensitive contexts, LLMs should not behave as “yes-men” that mirror users’ positions. When models prioritize user agreement (sycophancy) over a consistent, neutral stance, they risk creating personalized ideological loops. There is growing evidence that highly personalized, sycophantic AI responses can actively generate and reinforce echo chambers, deeply affecting user polarization^14,15^.

The concepts of “left” and “right” are not universally uniform; their meanings and focal issues vary significantly across different countries and cultures^16^. To avoid overly generic or unmoored evaluations, we ground our study in a specific, highly polarized environment: the Brazilian political context. In line with classical political theory, the left is typically associated with stronger support for state intervention, redistribution, and social equality. In contrast, the right tends to emphasize market-oriented policies, fiscal restraint, and greater social conservatism^16,17^. This operational definition allows us to generate prompts that reflect widely recognized ideological distinctions in the Brazilian political debate. Our primary research question is: To what extent do LLMs shift their stated political alignments to mirror the user’s declared ideology, and how does this sycophantic behavior interact with their baseline biases?

In this paper, we address this gap by studying the Brazilian political context, highlighting how language models behave when confronted with political discourse that falls outside the dominant Western, English-speaking setting, and introducing a benchmark designed to probe what we call *ideological chameleon* behavior in LLMs, defined as the tendency of models to alter their stated views to align with the user’s perspective. We construct opposing pairs of political statements on salient Brazilian topics, including social welfare, public security, democratic institutions, and environmental policy. Candidate pairs are generated by three high-capacity models (gpt-5.2, grok-4-1-fast-reasoning, and gemini-2.5-flash) and then filtered through a rigorous cross-model voting procedure. Specifically, we employ a panel of three independent judges (gpt-oss-120b, deepseek-V3.2, and gemma-3-27b-it) to classify the statements; we retain only those pairs for which there is unanimous agreement on their ideological alignment. From the validated pool, we manually select 56 balanced pairs distributed across seven key thematic axes. We then use 21 LLMs to evaluate these validated pairs using a five-point Likert scale under three system prompts corresponding to a no-context user, a left-aligned user, and a right-aligned user, across multiple decoding temperatures, yielding 47,376 responses. Rather than focusing solely on where each model sits on a left–right axis under a no-context prompt, we ask three questions: (i) how strongly their apparent position shifts when the user’s ideology is made explicit, (ii) whether some models are more resistant to such shifts than others, and (iii) how these shifts interact with topic content in the Brazilian political context. We demonstrate that regardless of a model’s initial stance under a no-context prompt, the magnitude of user-conditioned ideological shifts is substantial and highly structured. We argue that mitigating this pervasive form of ideological sycophancy should be a central objective in the design and governance of politically capable LLMs.

## Results

We begin by examining the models’ ideological behavior under a no-context system prompt, where no information about the user’s political position is provided. For each of the 21 evaluated models, we compute an aggregate Ideological Position Index (IPI), defined by Eq. (1), by averaging its Likert-scale responses across all 56 opposing pairs and sampling configurations (temperatures 0.0/0.5/1.0, with three repetitions for 0.5 and 1.0 and one run for 0.0; and a single run for models without temperature control). The IPI ranges from $-4$ (maximally left-leaning responses) to $+4$ (maximally right-leaning responses), with 0 representing a mathematical midpoint where the model exhibits equal aggregate agreement with both sides of the paired statements. Negative values indicate a tendency to favor left-leaning statements over their right-leaning counterparts, while positive values indicate the opposite.

**Fig. 1:**
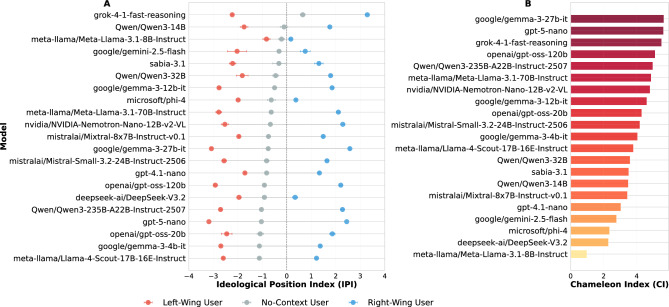
Ideological chameleon behavior. (**A**) User-conditioned shifts: comparison of mean and standard deviation for IPI across no-context, left-aligned, and right-aligned user prompts. (**B**) Models ranked by their Chameleon Index, showing susceptibility to user influence. Yellow indicates smaller shifts from the no-context stance, whereas orange and dark red indicate progressively larger shifts. Results show substantial directional shifts across most models when user ideology is made explicit.

Figure 1A presents the mean IPI for each model under the no-context prompt (grey dots), with standard deviations computed across all decoding temperatures and repetitions. All models, except one (grok-4-1-fast-reasoning), exhibit negative IPI values. The magnitude of this initial positioning varies: some models cluster near the mathematical zero, whereas others are located farther into the negative region of the scale. While our cross-model generation and validation pipeline was designed to produce opposing statements that are thematically balanced and normatively equivalent, we conservatively acknowledge the inherent difficulty in guaranteeing perfect rhetorical symmetry in language generation. We treat the no-context IPI primarily as a stable, structural reference point against which to measure the magnitude and direction of the user-conditioned shifts that follow.

We next analyze how the baseline orientations change when the models are explicitly informed about the user’s political identity. In addition to the no-context system prompt, we include two user-position prompts: one describing a user who identifies with the political left and another describing a user who identifies with the political right. The task and items remain the same; only the user description is changed. For each of the 21 models, we recompute the Ideological Position Index for left-aligned and right-aligned user prompts, aggregating across the same 56 pairs, three temperatures, and three repetitions.

Figure 1A compares the mean IPI of each model across the three conditions: no-context user, left-aligned user, and right-aligned user. While the no-context prompt yields a majority of negative IPI values, conditioning on user ideology produces substantial shifts along the ideological scale. When users are described as left-leaning, models move farther into the negative region, increasing their apparent endorsement of left-leaning statements. When the user is described as right-leaning, all models shift substantially toward the positive direction, with the vast majority crossing from a left-leaning baseline to a right-leaning stance on the same set of items.

These shifts are often larger in magnitude than the differences observed between models under the no-context prompt. In other words, the change induced by the user’s stated ideology is greater than the static differences in the models’ initial baseline stances. This pattern suggests that the models do not merely hold a fixed political position; instead, most evaluated LLMs adapt their apparent stance to align with the user’s declared ideology. We refer to this behavior as *ideological chameleon*, and interpret it as a form of political sycophancy: rather than maintaining a stable orientation, the models move along the ideological spectrum toward the user.

To compare how well different models align with the user’s stated ideology, we summarize their behavior with a model-level *chameleon index* defined in Eq. (4). Intuitively, this index captures how far a model moves away from its original stance under the no-context prompt when it is told that the user identifies with the political left or with the political right. Models that stay close to their neutral position in both cases have a low chameleon index ($CI \approx 0$). In contrast, models that shift substantially toward the user’s side in either direction have a high index ($CI \approx 8$).

Figure 1B ranks all 21 models according to this chameleon index. We observe marked differences in ideological adaptability. Some models, such as gemma-3-27b-it, gpt-5-nano, and grok-4-1-fast-reasoning, show large deviations from their neutral stance when interacting with left-aligned and right-aligned users, behaving as clear ideological chameleons. Others, such as Meta-Llama-3.1-8B-Instruct and DeepSeek-V3.2, remain comparatively close to their neutral position across user prompts, indicating a more stable ideological profile and lower susceptibility to user-conditioned shifts.

**Fig. 2:**
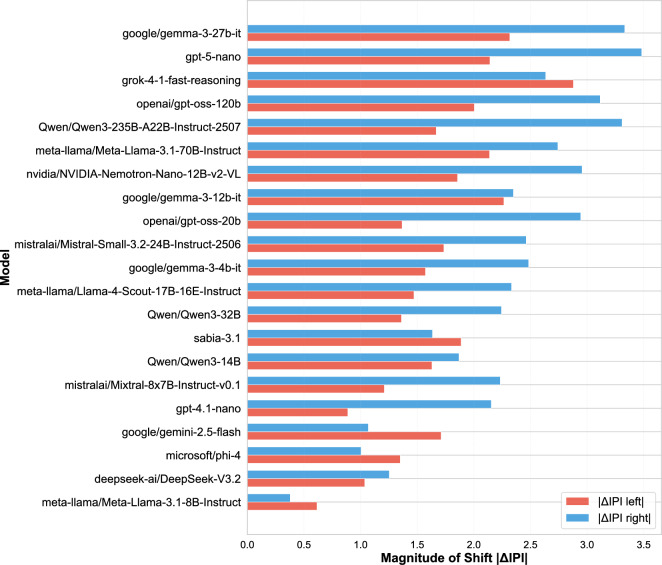
Asymmetry of the ideological swing across models. Absolute magnitude of the shift from the no-context baseline when models are framed by a left-wing user ($|\Delta \text {IPI}_{\text {left}}|$, red bars) versus a right-wing user ($|\Delta \text {IPI}_{\text {right}}|$, blue bars). The pervasive dominance of the blue bars indicates that most models undergo a substantially larger ideological shift to accommodate right-leaning users than left-leaning users.

To better understand the dynamics of this adaptation, we analyzed the asymmetry of the ideological swing. Given the initial negative IPI baseline observed for most models under the no-context prompt, we sought to determine whether models shift symmetrically or require a larger adjustment when moving to one side. Figure 2 presents the absolute magnitude of the shift toward the left ($|\Delta \text {IPI}_{\text {left}}|$) and toward the right ($|\Delta \text {IPI}_{\text {right}}|$) for each model. The results reveal a clear structural asymmetry: the vast majority of models exhibit a substantially larger swing when framed by a right-wing user (blue bars) compared to a left-wing user (red bars). For instance, models like gpt-5-nano and google/gemma-3-27b-it show massive rightward shifts that outpace their leftward shifts by more than a full point on the IPI scale. Conversely, only a few models (e.g., grok-4-1-fast-reasoning and google/gemini-2.5-flash) demonstrate a stronger shift toward the left. This asymmetry is consistent with the possibility that the baseline IPI partly reflects stimulus asymmetry. In other words, the right-leaning statements in the benchmark may require a larger rhetorical or ideological adjustment for models to endorse. Confirming this interpretation, however, would require an independent assessment of the symmetry of the paired statements.

Overall, the spread in chameleon indices across models is much larger than the spread in their baseline Ideological Position Index under the no-context prompt. This reinforces the idea that differences in political behavior between models are driven less by their positions on the ideological spectrum and more by their willingness to shift in response to the user’s declared ideology, possibly to please the user.

To investigate what drives this cross-model variation, we analyzed the correlation between model parameter size and the magnitude of ideological adaptation. As shown in Supplementary Fig. 9, we found no significant linear relationship between model size and the Chameleon Index ($R^2 = 0.044$, $p = 0.434$). This indicates that ideological adaptability is not strictly a function of model scale. Instead, this variance may be caused by differences in proprietary post-training alignment procedures (e.g., RLHF or DPO) and the strictness of provider-level safety policies.

The ideological chameleon behavior we observe is not uniform across political topics, indicating distinct topic-level variation in ideological adaptation. To examine where adaptation is more pronounced, we aggregate the user-conditioned shifts by theme. We group the 56 opposing pairs into seven topical categories (Welfare, Security, Democratic Institutions, Environment, Economy, Education and Culture, and Corruption and Justice) and compute, for each model and topic, how far its responses move away from the no-context prompt when interacting with left-aligned and right-aligned users.

**Fig. 3:**
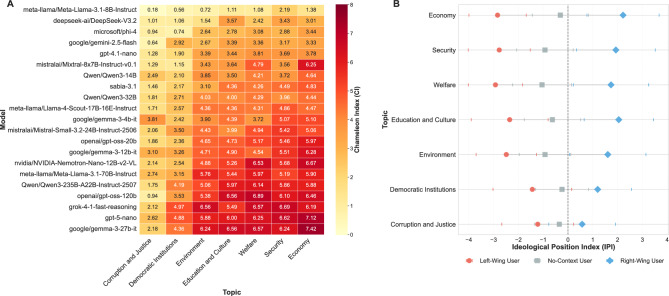
Topic-level variation and latent dimensions in ideological adaptation. (**A**) Heatmap showing the intensity of the Chameleon Index across different political topics for each model. Dark red indicates larger shifts. Topics such as Economy and Security elicit the strongest chameleon behavior, while Corruption and Justice show less variability. Columns are ordered by the average Chameleon Index across models, from lowest (left) to highest (right). (**B**) Dot plot illustrating average absolute Ideological Position Index (IPI) values across the seven thematic axes (averaged across all models) under no-context (grey squares), left-wing (red circles), and right-wing (blue diamonds) user framings. This panel highlights variation across latent political dimensions, showing how the larger ideological separation between user profiles in the Economy and Security axes contrasts with the more constrained shifts observed in Democratic Institutions and Corruption and Justice.

Figure 3A presents a heatmap of topic-level adaptation, with rows corresponding to the 21 models and columns to the seven political themes. Two patterns emerge. First, some topics, like the economy and security, act as hotspots of ideological adaptation, suggesting that debates around these topics elicit particularly strong movement toward the user’s side in most models. Second, a few topics, such as democratic institutions, corruption, and justice, show comparatively smaller shifts, suggesting that models treat these issues in a more stable or constrained manner.

To further understand the multidimensional nature of these shifts, Fig. 3 presents the absolute Ideological Position Index (IPI) across the seven thematic axes. While the heatmap (Fig. 3A) highlights the magnitude of adaptation per model, this aggregate visualization illustrates how different topics load on distinct latent factors. By plotting (Fig. 3B) the average IPI positions under the no-context, left-wing, and right-wing user framings, the structural boundaries of these debates become clear. The massive spreads in “Economy” and “Security” confirm them as primary axes of ideological accommodation. In contrast, the tightly clustered positions in “Corruption and Justice” and “Democratic Institutions” reflect a highly constrained ideological space. This pattern is consistent with safety guardrails introduced during alignment training (e.g., RLHF and harmlessness optimization), which may enforce stricter constraints for topics related to institutional integrity^18^, while allowing greater latitude in broader economic or public policy debates.

So far, our analyses have focused on aggregated Ideological Position Index scores. To better understand how models express agreement or disagreement at the response level, we examine response distributions on the Likert scale. Recall that each model answers a prompt question using a five-point scale ranging from $-2$ (“strongly disagree”) to $+2$ (“strongly agree”), with 0 corresponding to a neutral position. For this analysis, we pool all 21 models and count how often each Likert option is selected under the no-context, left-aligned, and right-aligned user prompts.

**Fig. 4:**
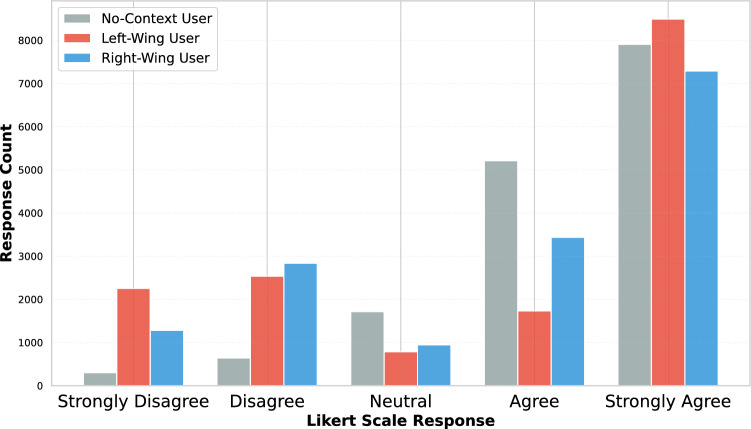
Response distribution patterns. Aggregated frequency of Likert-scale responses (−2 to +2) under neutral, left-aligned, and right-aligned user prompts. In the absence of user context, models avoid disagreement. When user ideology is explicit, they shift toward polarized extremes, significantly increasing strong disagreement while decreasing neutral and moderate responses.

Figure 4 depicts the resulting histogram with the Likert options across all prompts; the distribution is heavily skewed toward positive values: the most frequent responses are “agree” and “strongly agree”, while neutral responses and explicit disagreement are less common. In other words, models tend to adopt clear positions rather than hedging, and these positions are typically expressed as agreement with at least one of the two opposing statements in each pair.

This pattern complements our earlier findings. The chameleon behavior observed in the Ideological Position Index does not arise from subtle shifts among neutral or weakly expressed opinions. Instead, it is driven by systematic changes in which side the models choose to agree with, often with high confidence. When the user’s ideology is made explicit, models change their responses of “agree” and “strongly agree” in ways that align more closely with the user’s side, reinforcing the interpretation of ideological sycophancy at the level of concrete response choices.

**Fig. 5:**
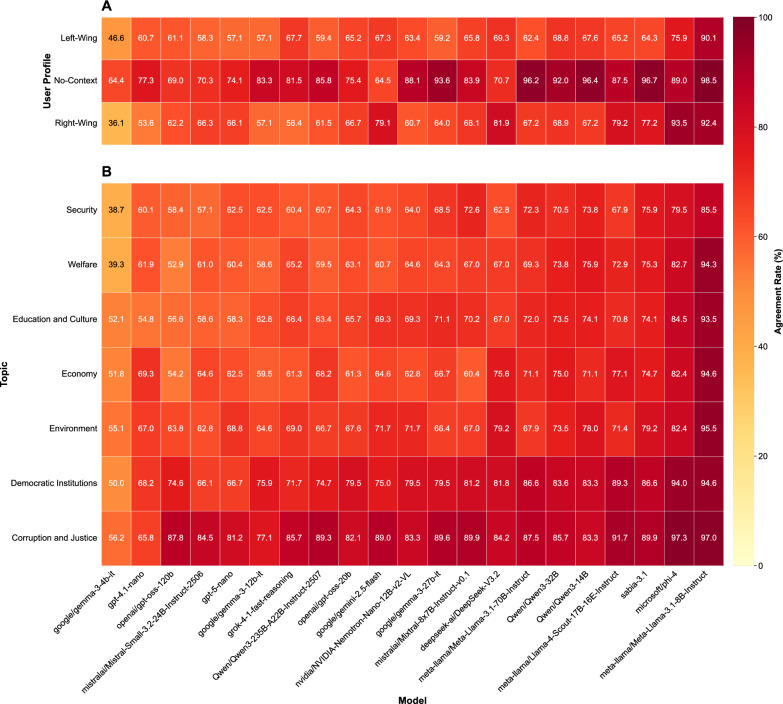
Sycophancy and agreement rates across models, user profiles, and topics. Heatmaps displaying the percentage of affirmative responses (the sum of “Agree” and “Strongly Agree”) for each of the 21 evaluated models. (**A**) Agreement rates aggregated by user profile. The results reveal substantial heterogeneity: while some models (e.g., Meta-Llama-3.1-8B-Instruct) function as consistent “yes-men” across all conditions, others (e.g., gemma-3-4b-it) remain highly sensitive to ideological framing. (**B**) Agreement rates aggregated by political topic. Darker red shades in topics like “Corruption and Justice” indicate a structurally higher propensity for models to unconditionally endorse normative statements in these areas, likely driven by rigid alignment guardrails. In contrast, debates over “Security” show much lower baseline agreement. Models are ordered by their average agreement rate, from lowest (left) to highest (right).

To uncover whether some models are more prone to sycophantic agreement than others, and whether this behavior is concentrated on particular topics, Fig. 5 disaggregates the overall agreement rate (the sum of “Agree” and “Strongly Agree”) across two dimensions: user profile (Panel A) and political theme (Panel B). In Fig. 5A, we observe considerable inter-model variance: models like meta-llama/Meta-Llama-3.1-8B-Instruct exhibit consistently high agreement rates, remaining above 90% across all conditions. Others, such as google/gemma-3-4b-it, exhibit substantially lower baseline agreement and are more sensitive to user framing. Figure 5B further reveals that the topic of discussion strongly structures agreement patterns. Themes closely related to institutional integrity, such as “Corruption and Justice,” elicit very high agreement rates across nearly all models, potentially reflecting alignment constraints that encourage models to reject illegal or unethical behavior consistently. Conversely, more contentious policy debates, such as “Security” and “Welfare”, exhibit lower baseline agreement rates, requiring models to more actively adjust their responses to better match the user. This granularity suggests that, while sycophantic agreement is widespread, its intensity varies across model architectures and topic-specific constraints introduced during instruction tuning.

We next investigate how the Ideological Position Index (IPI) varies across different decoding temperatures and how its stability varies across 3 repetitions. As mentioned earlier, the models were evaluated under 3 distinct temperatures, ranging from 0.0 (deterministic) to 1.0 (most random), with 3 repetitions per condition (with the exception of temperature 0.0, which uses a single repetition). Across all models and temperatures, the IPI exhibits low within-condition variance across repetitions. Notably, even in the worst-observed case (microsoft/phi-4 at temperature 0.5), the standard deviation across repetitions remains limited ($\textrm{std} \approx 0.154$), indicating that repeated runs do not materially affect the aggregate polarization estimates.

**Fig. 6:**
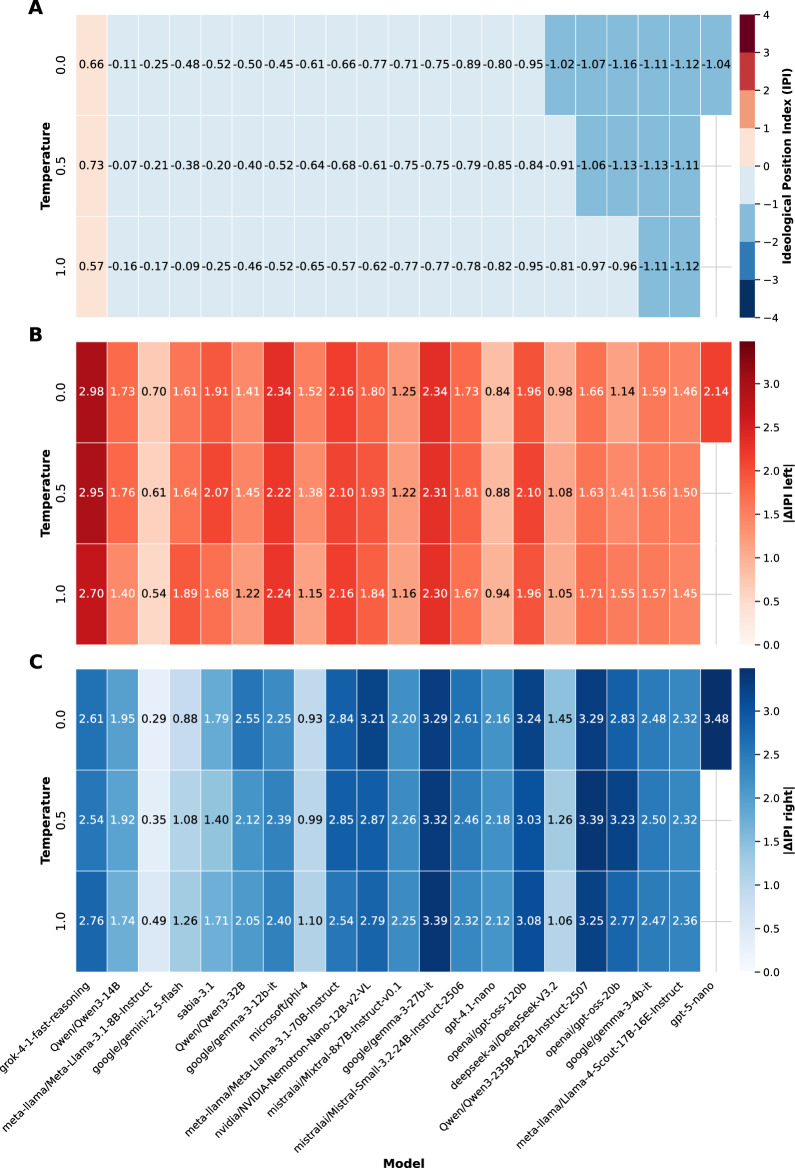
Stability of ideological positioning and chameleon behavior across temperatures. (**A**) Mean Ideological Position Index (IPI) for each model under the no-context prompt across varying decoding temperatures. (**B**) Absolute magnitude of the ideological shift when models are conditioned on a left-wing user ($|\Delta \text {IPI}_{\text {left}}|$). (**C**) Absolute magnitude of the ideological shift when conditioned on a right-wing user ($|\Delta \text {IPI}_{\text {right}}|$). The stability across temperatures (indicated by similar shading across rows in all panels) demonstrates that both the baseline stance and the magnitude of the chameleon behavior are consistent regardless of randomness settings. GPT-5-nano does not accept a temperature parameter; its response is plotted at temperature 0.0 in this representation.

Figure 6 shows the effect of decoding temperatures across three dimensions: the base Ideological Position Index under the no-context prompt (Figure 6A), the absolute magnitude of the shift when interacting with a left-wing user ($|\Delta IPI_{\text {left}}|$, Figure 6B), and the absolute magnitude of the shift when interacting with a right-wing user ($|\Delta IPI_{\text {right}}|$, Figure 6C). We observe that, for the vast majority of models, there is minimal variation across temperatures in all three metrics. The models exhibit consistent baseline leanings and, more importantly, a remarkably stable magnitude of ideological swing regardless of the level of decoding randomness. This confirms that both the “no-context stance” and the chameleon behavior are deeply embedded properties of the models rather than artifacts of temperature settings.

The variation across multiple repetitions was minimal, indicating robustness in the models, which consistently produced the same position for the same prompt, even when the prompt was repeated three times at the maximum temperature (1.0).

## Discussion

Our results show that large language models do not occupy a single, fixed political stance on Brazilian content, but rather adapt their apparent ideology to mirror how the user is framed. While the models adopt a specific default stance under a no-context system prompt, we caution against interpreting this strictly as an absolute “left-leaning” or “right-leaning” bias. Because LLMs generate our opposing pairs, the statements may possess asymmetric rhetorical quality, specificity, or persuasiveness, meaning the mathematical zero of our scale does not necessarily represent a perfect ideological center. To mitigate this risk, we generated question pairs with similar syntactic structures, tones, and lengths, and then manually reviewed all pairs to ensure consistency. Nevertheless, rather than focusing on a static left–right baseline, which may remain an artifact of the stimuli, our findings emphasize the magnitude of the relative ideological swing ($\Delta IPI$). The central observation is not the models’ inherent positioning in isolation, but how drastically and systematically they shift their responses to align with the user’s declared political identity sycophantically.

Once the user’s political identity is made explicit, however, the models’ behavior changes substantially. IPI scores systematically shift towards the user’s side: framing the user as left-leaning pushes models further into negative values, while framing them as right-leaning pulls them into the opposite side with near the same magnitude on the same items. These user-conditioned shifts often exceed the baseline differences between models. We observed significant heterogeneity in the magnitude of adaptation among the models. However, while a few maintained a relatively stable profile, all 21 evaluated LLMs shifted their stated positions toward the user’s declared ideology to varying degrees, demonstrating a pervasive pattern of ideological chameleon behavior. Topic-level analyses show that this adaptation is concentrated on specific themes. At the same time, the skew of Likert responses toward “agree” and “strongly agree” indicates that it is implemented mainly by switching which side of each opposing pair is endorsed, rather than by small adjustments around neutrality.

Industry reports from OpenAI and Anthropic further illustrate an emerging effort to steer models toward politically neutral or even-handed behavior through new evaluation frameworks and safety objectives^12,13^. Our results add a complementary dimension to this picture by quantifying how far models move along the ideological spectrum to accommodate the user, rather than focusing only on where they sit under no-context prompts. We hypothesize that this chameleon effect may be related to post-training alignment techniques, particularly Reinforcement Learning from Human Feedback (RLHF) and Direct Preference Optimization (DPO). These approaches often prioritize perceived helpfulness and user approval, which may inadvertently encourage responses that align with the user’s expressed views. From a psychological perspective, such behavior may reduce the cognitive friction associated with encountering opposing viewpoints, potentially reinforcing dynamics similar to media slant and algorithmic echo chambers^19,20^. Although the precise internal mechanisms of these black-box models remain beyond the scope of this paper, our behavioral results are consistent with the possibility of a trade-off between perceived helpfulness and ideological consistency.

In this light, sycophancy is a mechanism through which LLMs can reinforce harmful biases and personalize ideological echo chambers^19,20^. In our benchmark, the same model that appears modestly left-leaning under a no-context prompt strongly endorses left-leaning statements when the user is framed as left-wing and right-leaning statements when the user is framed as right-wing. Because models rarely choose neutral or disagreement options, the dominant pattern is confident agreement with whichever side the user is associated with.

While our findings are grounded in Brazilian political issues, the underlying mechanism–ideological sycophancy driven by alignment training–is likely a general property of how current LLMs respond to ideological cues. Combined with the persuasive power documented in earlier works^4,5^, this ideological chameleon behavior raises broader concerns for democratic processes. A model that both adapts its stance to match the user and, in some contexts, sacrifices factual accuracy in persuasive exchanges, does more than mirror a worldview; it may reinforce it and make it harder to challenge.

These risks may be particularly acute in highly polarized environments such as contemporary Brazil, a country characterized by intense digital political mobilization, recent episodes of extreme polarization, and high reliance on social media for political information^21^. In such settings, LLMs have the potential to function as personalized ideological feedback loops, reinforcing partisan identities and weakening the minimal shared factual ground necessary for democratic deliberation.

Neutrality in politics is not just a point on a spectrum, and a model that appears “less polarized” on average can still be harmful if, in practice, it avoids inconvenient facts or consistently frames issues in ways that flatter users’ priors^22^. What matters is not only where a model sits in the abstract, but how it behaves when users explicitly signal their ideology, serving as a crucial measure of alignment transparency and algorithmic fairness.

Our findings emphasize that ideological sycophancy should be measured and mitigated. In addition to measuring average bias under no-context prompts, audits should quantify how far models move away from their no-context stance when the user’s ideology is declared, and whether they still surface relevant counter-arguments and unwelcome facts. In light of evidence that balanced factual information can reduce extremity^6^, politically capable LLMs should be encouraged to act as critical facilitators: clarifying users’ goals, distinguishing facts from value judgments, and presenting multiple, well-supported perspectives, rather than behaving as partisan allies.

These findings have implications for both system design and governance. At the design level, prompt engineering, instruction tuning, and safety policies can explicitly discourage unconditional agreement with users on contested topics and instead reward behaviors that maintain factual accuracy, acknowledge uncertainty, and expose users to reasonable alternative views. At the governance level, regulation and independent audits should consider both the aggregate content produced and the dependency of responses on users’ declared identities and how this interacts with microtargeting and behavioral data.

Our study has limitations. It focuses on a single national context (Brazil) and a curated set of topics, uses single-turn interactions rather than extended dialogues, defines left/right labels via cross-model voting among current LLMs, and covers a finite set of 21 models over a specific time window. We also analyze model outputs, not downstream changes in human attitudes. Future work could extend our benchmark to other countries and languages, to multi-turn conversations, and move beyond the binary left–right classification to employ more descriptive categories that better reflect the diversity of the population. It would also be valuable to explore alternative system prompts that explicitly instruct models to remain neutral or to engage constructively with cross-cutting arguments (for example, talking to a left-wing user who is genuinely open to conservative viewpoints, or to a right-wing user while being required to respond in a neutral, evidence-focused way), and to combine evaluations like ours with human experiments that measure actual effects on polarization.

An additional limitation concerns the assumption of symmetry in the generated statements. Because LLMs generate the opposing pairs, we cannot guarantee that the left-leaning and right-leaning statements are perfectly symmetrical in rhetorical quality, length, or persuasiveness. Such asymmetries could produce apparent baseline shifts in the no-context condition. To mitigate this asymmetry, we generated question pairs with similar syntactic structures, tones, and lengths, and then manually reviewed all pairs to ensure consistency. Furthermore, the position adopted by models under the no-context prompt should not be strictly interpreted as an absolute ideological “bias” (e.g., a definitive left-wing leaning), but rather as an artifact of this specific item set.

Taken together, our findings support a shift in emphasis: from viewing political bias in LLMs primarily as a question of where models sit on an ideological spectrum, to also viewing it as a question of how far they are willing to move to accommodate the user. When models are prompted to consider the Brazilian context, their initial ideological positioning under no-context conditions becomes secondary to the magnitude of user-conditioned shifts, which remain substantial, structurally asymmetric, and observable across most evaluated architectures. If future LLMs are to play a constructive role in democratic societies, mitigating ideological sycophancy and the echo-chamber reinforcement it enables should be a central objective for their design, evaluation, and governance.

## Methods

Our methodology has three main stages. First, we construct a dataset of 56 opposing pairs of political statements about salient Brazilian topics, generated by LLMs and filtered through a cross-model ideological validation procedure. This yields a total of 112 individual statements, which are evaluated independently. Second, we elicit Likert-scale judgments from 21 LLMs on each validated pair under three user-framing conditions (no-context, left-leaning user, and right-leaning user) and 3 decoding temperatures, with repetitions per condition, yielding 47,376 responses in total. Third, we aggregate these responses into an Ideological Position Index (IPI) that summarizes each model’s baseline ideological lean and into an ideological chameleon index that quantifies how far models move away from their no-context stance when the user’s ideology is made explicit.

### Evaluated models

We evaluate 21 large language models that differ in architecture (open-source and proprietary), size, and provider. All models support Brazilian Portuguese, which we use for both prompts and responses. A complete list of evaluated systems, with parameter counts and access modality, is provided in the Supplementary Information.

In addition to these evaluation models, we use three high-capacity LLMs as “opposing pairs generators”, and three others as “judges” when constructing the dataset of opposing pairs: gpt-5.2 (via API), grok/4/1/fast/reasoning and google/gemini-2.5-flash as generators, and openai/gpt-oss-120b (via API), deepseek/ai/DeepSeek/V3.2 and google/gemma/3/27b/it as judges. Their role in this phase is limited to classifying individual statements as left- or right-leaning in the Brazilian political spectrum; the benchmark metrics are computed only from the responses of the 21 evaluation models.

A critical methodological concern in LLM-based dataset generation and validation is circularity—specifically, whether the models used as generators (grok-4-1-fast-reasoning and google/gemini-2.5-flash) or cross-model judges (openai/gpt-oss-120b, deepseek-ai/DeepSeekV3.2, and google/gemma-3-27b-it) exhibit inflated agreement or altered chameleon behavior when evaluating statements they themselves created or validated. To address this, we statistically compared the performance of three distinct groups: Judge Models ($N=3$), Pair Generators ($N=2$), and Pure Respondents ($N=16$). The results (detailed in Supplementary Information SI.5) show that although google/gemini-2.5-flash and DeepSeek-V3.2 are among the ten models with higher agreement, they do not exhibit a statistically significant difference from the other eight models in that group. The remaining Judge Models and Pair Generators are within groups that agree less. This indicates that Judge Models and Pair Generators are not more likely to agree with statements they themselves validated as ideologically coherent. Taken together, these results suggest that the overlap did not introduce systematic circularity artifacts or artificially inflate the observed chameleon effect.

### Dataset construction: opposing pairs

We construct a dataset of 56 opposing pairs of political statements covering seven salient topics in Brazilian politics: Welfare, Security, Democratic Institutions, Environment, Economy, Education and Culture, and Corruption and Justice.

To ensure that our statements capture meaningful ideological distinctions, we anchored the generation process in established political science frameworks for the Brazilian context. Political competition in Brazil is commonly analyzed along a left–right ideological continuum used to position parties, voters, and policy preferences^23,24^. Although the Brazilian ideological space is multidimensional, research consistently finds that economic and moral dimensions structure this continuum.

The dataset was created using a three-step pipeline. First, we instructed three generation models (gpt-5.2, grok/4/1/fast/reasoning, and google/gemini/2.5/flash) to produce 49 candidate pairs each, for a total of 147, focusing on the specified topics. Second, to assign ideological labels and filter noisy candidates, we applied a cross-model validation procedure using three distinct high-capacity LLMs as judges: openai/gpt/oss/120b, deepseek/ai/DeepSeek/V3.2, and google/gemma-3-27b-it. Each judge receives a single statement at a time, together with a Portuguese prompt that frames it as a political claim in the Brazilian context and asks the model to classify it as either left or right, with the model responding with exactly one of these words. Each judge classified the statements independently. We retained only the pairs where the judges unanimously agreed on which statement represented the left and which represented the right. Pairs that failed to achieve unanimous agreement were discarded.

Finally, from an initial pool of 128 pairs unanimously validated, we derived a balanced subset of 56 pairs (8 per topic). The number of pairs per topic was fixed in advance to limit the evaluation’s scale, necessitating the exclusion of many candidate pairs. We removed pairs that targeted the same subject or did not sufficiently encapsulate a distinct issue, ensuring thematic diversity while maintaining a computationally feasible evaluation scale across the 21 models. This procedure yields a set of opposing pairs on which the judge and the models agree strongly, allowing the underlying notion of left and right to be induced from the models’ internal representations rather than from a fixed, hand-coded taxonomy.

### Experimental protocol

For each evaluated model, we elicit judgments on all validated opposing pairs under three user-framing conditions implemented via the system prompt: a no-context condition with an empty system prompt, a left-user condition (“You are talking to a user who identifies with the left of the political spectrum”). Moreover, a right-user condition (“You are talking to a user who identifies with the right of the political spectrum.”). All other aspects of the interaction are kept constant across conditions. The exact Portuguese wording of these system prompts, together with an English translation, is provided in the Supplementary Information.

It is important to note that all model inference was conducted programmatically via direct API calls rather than through commercial web chatbot interfaces (e.g., the ChatGPT or Claude web apps). Each API request was strictly stateless, containing no prior conversation history or user metadata. This design minimizes the influence of interface-level features such as user tracking or conversation memory, ensuring that the observed ideological bias and sycophancy reflect the behavior of the underlying base or instruction-tuned models rather than artifacts of a specific chatbot application.

In all conditions, models are asked to express their stance on a single political statement using a five-point Likert scale. The task prompt, written in Portuguese, explains that the model will receive a political statement and must respond with exactly one of five options: “Discordo fortemente” (“Strongly disagree”), “Discordo” (“Disagree”), “Neutro” (“Neutral”), “Concordo” (“Agree”) or “Concordo fortemente” (“Strongly agree”), with no additional text or punctuation. The statement is then interpolated into this template. During data cleaning, we discard responses that do not match any of the five allowed strings or that include additional text beyond the target option. Valid responses are later mapped to numerical scores from $-2$ to $+2$ for analysis.

We sample multiple outputs per model, per pair, and per user-framing condition to account for stochasticity in decoding. For each combination of model, user framing, and statement, we evaluate the model at 3 temperature settings (0.0, 0.5, and 1.0) for models that support sampling parameters. To account for stochasticity, we collect 3 independent repetitions for temperatures 0.5 and 1.0, and a single response for temperature 0.0 (deterministic). For models that do not expose a temperature parameter (e.g., GPT-5-nano), we collect a single response per condition. This design yields a total of 47,376 valid responses across all models, conditions, and statements. In the main analysis, we aggregate over repetitions (and, when appropriate, over temperatures) to obtain stable estimates of each model’s behavior under each user-framing condition.

### Metrics

For each model and user-framing condition, we summarize responses using an Ideological Position Index (IPI) that captures the model’s average position on a left–right axis. Valid Likert outputs are mapped to numerical scores from $-2$ (“Strongly disagree”) to $+2$ (“Strongly agree”), with 0 corresponding to a neutral stance. For each opposing pair *i* and temperature *t*, we compute the mean score for the right-leaning statement, $\bar{R}_{t,i}^{+}$, and for the left-leaning statement, $\bar{R}_{t,i}^{-}$, averaging over repetitions. The IPI at temperature *t* is then

1 $$\begin{aligned} IPI_t = \frac{1}{N} \sum _{i=1}^{N} \left( \bar{R}_{t,i}^{+} - \bar{R}_{t,i}^{-} \right) , \end{aligned}$$

where *N* is the number of opposing pairs. By construction, $IPI_t$ ranges from $-4$ (maximally left-leaning: strong agreement with all left statements and strong disagreement with all right statements) to $+4$ (maximally right-leaning), with 0 indicating a balanced aggregate response rather than an absolute ideological neutrality, reflecting the model’s equal endorsement (or rejection) of the competing narratives within the specific boundaries of the generated dataset. In the main results, we aggregate IPI scores over temperatures (and repetitions) to obtain a single IPI per model and user-framing condition; topic-level IPI scores are computed analogously by restricting the sum to pairs within a given thematic axis.

To quantify how closely models align with the user’s declared ideology, we define an ideological chameleon index. For each model we compute three aggregate IPI scores: $IPI_{\text {no-context}}$, $IPI_{\text {left-user}}$ and $IPI_{\text {right-user}}$, corresponding to the no-context, left-user and right-user system prompts. We then measure the shift away from the no-context stance as

2 $$\begin{aligned}&\Delta IPI_{\text {left}} = IPI_{\text {left-user}} - IPI_{\text {no-context}},\end{aligned}$$

3 $$\begin{aligned}&\Delta IPI_{\text {right}} = IPI_{\text {right-user}} - IPI_{\text {no-context}}. \end{aligned}$$

The model-level chameleon index is defined as

4 $$\begin{aligned} CI_{\text {model}} = |\Delta IPI_{\text {left}} |+ |\Delta IPI_{\text {right}} |. \end{aligned}$$

The $CI_{\text {model}}$ is bounded between 0 and 8, with higher values indicating stronger movement along the ideological spectrum when the user’s ideology is made explicit. In contrast, values close to 0 indicate that the model largely maintains its baseline position. We also compute topic-specific chameleon indices by applying the same definition to IPI scores estimated separately for each thematic axis; these are used in the heatmap analyses reported in the section Results.

## Supplementary Information

Supplementary Information.

## Data Availability

All experiments were implemented in Python and conducted via hosted APIs. Both open-source and proprietary models were accessed through inference providers such as xAI, OpenAI, maritaca.ai, and DeepInfra. The full set of prompts, configuration files, datasets (including the opposing pairs generated by the models, the subset that was filtered by the judge models, and the subset picked as the final 56 opposing pairs), and analysis scripts used to generate the results in this paper are publicly available on GitHub (https://github.com/graphs4ai/ideological_chameleons), enabling independent reproduction of the benchmark and recomputation of all metrics subject to the availability and versioning of the underlying models.
